# On the Role of Salt in Immunoregulation and Autoimmunity

**DOI:** 10.31138/mjr.32.1.3

**Published:** 2020-04-30

**Authors:** Athanasios Mavropoulos

**Affiliations:** Department of Rheumatology and Clinical Immunology, Faculty of Medicine, School of Health Sciences, University of Thessaly, Larissa, Greece

**Keywords:** Salt, Diet, Autoimmunity, Tregs, FoxP3, cytokines

The incidence of autoimmune rheumatic diseases (ARD) has markedly increased over recent decades, supporting the idea that changes in environmental factors such as infections, water/air contaminants and dietary habits may play a role in the aetiopathogenesis of these diseases, a notion also supported by studies investigating the incidence of such diseases in monozygotic twins.^1–3^ Salt (sodium chloride; NaCl) is amongst the most common dietary constituents that was increased in our diet, as a consequence of the consumption of processed foods.^4^ Although a vital nutrient and a major constituent of mammalian cell physiology, *in vitro* experimental observations have indicated that immune cells, when exposed to hypertonic saline produce pro-inflammatory cytokines, release reactive oxygen species and participate in the activation of the inflammasome.^5–7^

*In vivo*, the role of high salt dietary intake in the emergence/ exacerbation of autoimmunity has been extensively studied in several autoimmune animal models including the Collagen Induced Arthritis (CIA) model for rheumatoid arthritis (RA), the Experimental Autoimmune Encephalomyelitis (EAE) model for multiple sclerosis (MS), MRL/lpr mouse model for lupus nephritis and dextran sulfate sodium induced colitis models for Crohn’s disease.^8^ The data from animal models ubiquitously revealed a detrimental role of high sodium intake in reversing the suppressive effects of Regulatory T cells (Tregs) and promoting a cellular shift toward T-helper (Th)-1 and Th17 pro-inflammatory phenotypes.^9,10^ Moreover, Th17 cells overexpressed IL-17A and IL-23R, had increased phosphorylation of p38 mitogen-activated protein kinase (MAPK), induced expression of the osmosensitive nuclear factor of activated T cells 5 (NFAT5) and its target serum/glucocorticoid-regulated kinase 1 (SGK1).^11,12^ SGK1 has been implicated in polarization toward the Th17 phenotype, which deteriorates immune homeostasis and promotes pathogenesis in diseases such as RA, psoriatic arthritis, systemic lupus erythematosus, MS, autoimmune colitis, and transplant rejection. Clinical and epidemiological studies, although scarce, are in accord with these findings supporting the notion that high sodium intake presents a notably positive correlation with increased disease activity in MS and RA patients. Excessive sodium intake is actually associated with increased risk of RA disease emergence, particularly in smokers.^13,14^ Studies in patients with MS have also demonstrated an association between excessive salt in-take and a higher number of flares.^15^ These data suggest that salt truly stimulates certain pathological immunological processes and strengthen a directly linked influence of dietary habits on the development and progression of autoimmune diseases.^8^

Until recently, investigators have studied extensively and placed all their hopes on the thymus-derived, naturally occurring CD4+Foxp3+ (nTregs) which play an essential role in immunologic homeostasis and the prevention of autoimmune diseases.^16^ However, hype soon turned into disappointment when the first experimental results came out showing that high sodium concentrations lead to Foxp3 downregulation and reduction of the immunosuppressive function in the presence of pro-inflammatory cytokines *in vitro a*nd *in vivo.*^17^ Moreover, in the presence of high salt, the phenotype of these Tregs was highly unstable, and especially in inflammatory conditions, nTregs converted into other T effector cell subsets, such as Th1, Th2, Th17, and Tfh cells.^18^

However, a recent study from Luo et al.^19^ provided a glimmer of hope into what was previously thought a closed case, under the verdict that Tregs are unable to reverse or mediate suppression under high dietary salt conditions. Luo et al. actually showed that high salt does not affect the characteristics of Transforming growth factor β (TGF-β)- induced regulatory T cells (iTregs). iTregs were stable and functional in the presence of high salt during autoimmune responses.

In addition to the nTreg subset, CD4+ Treg cells can be induced in the periphery outside of thymus (peripheral Treg) or from conventional non-Treg cells in the presence of TGF-β and interleukin (IL)-2 with appropriate antigens (iTreg).^20^ iTreg subsets exhibit similarities and differences with their naturally occurring thymus derived counterparts, for instance they both express Foxp3. However, the iTreg subset also displays the substantial difference that it can be stable under inflammatory conditions. Whether high levels of dietary salt also affect the phenotype and function of the iTreg subset was previously unknown.

Luo et al. convincingly demonstrated that high salt did not influence the development, differentiation, and functional activities of iTreg, but affected Foxp3 stability and function of nTreg *in vitro* and *in vivo*. The iTreg differentiation was conducted with a standard protocol as previously described in the presence of different concentrations of NaCl (20–40 mM) with different time points. The addition of TGF-β greatly enhanced Foxp3 induction, and more than 90% of naive CD4+ cells became CD4+CD25+-Foxp3+ cells. Interestingly, high salt did not diminish but even slightly increased Foxp3 induction. Conversely, high salt reduced the Foxp3 expression and functionality of nTreg subset *in vitro* and *in vivo*.

Furthermore, high salt did not significantly alter the transcription profiling of the iTreg gene signatures. More specifically they performed RNA sequencing (RNA-seq) analyses which showed that high levels of dietary salt did not significantly change the gene profiles related to Treg and inflammatory cell signatures. They provided evidence for an additional difference between nTreg and iTreg subsets, and implied that iTregs can easily adapt to environmental factors. This subset may therefore have advantages in treating patients with autoimmune diseases in the future.

Regarding phenotypic and functional stability, Luo et al. reported that physiologically elevated levels of sodium chloride in vitro affected neither the differentiation nor function of the iTreg subset. The iTreg subset that had differentiated in vitro was resistant to Th17 and Th1 cell conversion in the presence of high levels of sodium chloride. As high salt promotes the nTreg subset to secrete interferon gamma (IFNγ) they also analyzed whether the iTreg subset began to express IFNγ under the influence of high salt. The iTreg subset did not produce proinflammatory cytokines including IFN-γ and IL-17A and was almost completely resistant to Th1 and Th17 conversion in the presence of high salt. Moreover, high salt actually increased iTreg proliferation.

Luo et al. also tested whether the iTreg subset is still resistant to high salt *in vivo*. They used a colitis model to assess the effects of high salt-treated iTreg and showed that these iTregs can also markedly alleviate typical pathological features as normal iTreg. iTregs could also strongly inhibit T cell-induced colitis, and high salt did not compromise the in vivo function. Furthermore, co-transfer of both iTreg and iTreg that had been pretreated with high salt significantly inhibited the Th17 and Th1 production in spleens and Th17 production in mesenteric lymph nodes.

They also extended their observations from mouse cells to human cells. Although a difference between mouse and human iTreg cell development appears to exist, recent studies converge to the opinion that the addition of all-trans retinoic acid universally facilitates induction of human iTreg in the presence of TGF-β.^21,22^ Using this approach, they concluded that the human naive CD4+ cells could successfully differentiate into Foxp3+ cells with potent in vitro suppression. Interestingly, upon high salt exposure, neither the differentiation nor function of human iTreg cells was compromised.

Therefore, the human iTreg subset are also stable and functional in the presence of high salt. These findings provide long sought evidence that iTreg have different biological features from nTreg and represent a Treg subset with greater potential for clinical utility in patients with autoimmune diseases, in which the complicated role of environmental factors, including diet, must be considered.
